# Image Analysis for MRI Based Brain Tumor Detection and Feature Extraction Using Biologically Inspired BWT and SVM

**DOI:** 10.1155/2017/9749108

**Published:** 2017-03-06

**Authors:** Nilesh Bhaskarrao Bahadure, Arun Kumar Ray, Har Pal Thethi

**Affiliations:** ^1^School of Electronics Engineering, KIIT University, Bhubaneswar, Odisha, India; ^2^Department of Electronics & Telecommunication Engineering, Lovely Professional University, Jalandhar, Punjab, India

## Abstract

The segmentation, detection, and extraction of infected tumor area from magnetic resonance (MR) images are a primary concern but a tedious and time taking task performed by radiologists or clinical experts, and their accuracy depends on their experience only. So, the use of computer aided technology becomes very necessary to overcome these limitations. In this study, to improve the performance and reduce the complexity involves in the medical image segmentation process, we have investigated Berkeley wavelet transformation (BWT) based brain tumor segmentation. Furthermore, to improve the accuracy and quality rate of the support vector machine (SVM) based classifier, relevant features are extracted from each segmented tissue. The experimental results of proposed technique have been evaluated and validated for performance and quality analysis on magnetic resonance brain images, based on accuracy, sensitivity, specificity, and dice similarity index coefficient. The experimental results achieved 96.51% accuracy, 94.2% specificity, and 97.72% sensitivity, demonstrating the effectiveness of the proposed technique for identifying normal and abnormal tissues from brain MR images. The experimental results also obtained an average of 0.82 dice similarity index coefficient, which indicates better overlap between the automated (machines) extracted tumor region with manually extracted tumor region by radiologists. The simulation results prove the significance in terms of quality parameters and accuracy in comparison to state-of-the-art techniques.

## 1. Introduction

In recent times, the introduction of information technology and e-health care system in the medical field helps clinical experts to provide better health care to the patient. This study addresses the problems of segmentation of abnormal brain tissues and normal tissues such as gray matter (GM), white matter (WM), and cerebrospinal fluid (CSF) from magnetic resonance (MR) images using feature extraction technique and support vector machine (SVM) classifier [1, 2].

The tumor is basically an uncontrolled growth of cancerous cells in any part of the body, whereas a brain tumor is an uncontrolled growth of cancerous cells in the brain. A brain tumor can be benign or malignant. The benign brain tumor has a uniformity in structure and does not contain active (cancer) cells, whereas malignant brain tumors have a nonuniformity (heterogeneous) in structure and contain active cells. The gliomas and meningiomas are the examples of low-grade tumors, classified as benign tumors and glioblastoma and astrocytomas are a class of high-grade tumors, classified as malignant tumors.

According to the World Health Organization and American Brain Tumor Association [3], the most common grading system uses a scale from grade I to grade IV to classify benign and malignant tumor types. On that scale, benign tumors fall under grade I and II glioma and malignant tumors fall under grade III and IV glioma. The grade I and II glioma are also called low-grade tumor type and possess a slow growth, whereas grade III and IV are called high-grade tumor types and possess a rapid growth of tumors. If the low-grade brain tumor is left untreated, it is likely to develop into a high-grade brain tumor that is a malignant brain tumor. Patients with grade II gliomas require serial monitoring and observations by magnetic resonance imaging (MRI) or computed tomography (CT) scan every 6 to 12 months. Brain tumor might influence any individual at any age, and its impact on the body may not be the same for every individual.

The benign tumors of low-grade I and II glioma are considered to be curative under complete surgical excursion, whereas malignant brain tumors of grade III and IV category can be treated by radiotherapy, chemotherapy, or a combination thereof. The term malignant glioma encompasses both grade III and IV gliomas, which is also referred to as anaplastic astrocytomas. An anaplastic astrocytoma is a mid-grade tumor that demonstrates abnormal or irregular growth and an increased growth index compared to other low-grade tumors. Furthermore, the most malignant form of astrocytoma, which is also the highest grade glioma, is the glioblastoma. The abnormal fast growth of blood vessels and the presence of the necrosis (dead cells) around the tumor are distinguished glioblastoma from all the other grades of the tumor class. Grade IV tumor class that is glioblastoma is always rapidly growing and highly malignant form of tumors as compared to other grades of the tumors.

To detect infected tumor tissues from medical imaging modalities, segmentation is employed. Segmentation is necessary and important step in image analysis; it is a process of separating an image into different regions or blocks sharing common and identical properties, such as color, texture, contrast, brightness, boundaries, and gray level. Brain tumor segmentation involves the process of separating the tumor tissues such as edema and dead cells from normal brain tissues and solid tumors, such as WM, GM, and CSF [4] with the help of MR images or other imaging modalities [5–8].

In this study, different magnetic resonance imaging (MRI) sequence images are employed for diagnosis, including T1-weighted MRI, T2-weighted MRI, fluid-attenuated inversion recovery- (FLAIR) weighted MRI, and proton density-weighted MRI. The detection of a brain tumor at an early stage is a key issue for providing improved treatment. Once a brain tumor is clinically suspected, radiological evaluation is required to determine its location, its size, and impact on the surrounding areas. On the basis of this information the best therapy, surgery, radiation, or chemotherapy, is decided. It is evident that the chances of survival of a tumor-infected patient can be increased significantly if the tumor is detected accurately in its early stage [9]. As a result, the study of brain tumors using imaging modalities has gained importance in the radiology department.

The rest of the paper is organized as follows: Section 2 presents the related works, Section 3 presents the materials and methods with the steps used in the proposed technique, Section 4 presents the results and discussion, Section 5 presents the comparative analysis, and finally Section 6 contains the conclusions and future work.

## 2. Related Works

Medical image segmentation for detection of brain tumor from the magnetic resonance (MR) images or from other medical imaging modalities is a very important process for deciding right therapy at the right time. Many techniques have been proposed for classification of brain tumors in MR images, most notably, fuzzy clustering means (FCM), support vector machine (SVM), artificial neural network (ANN), knowledge-based techniques, and expectation-maximization (EM) algorithm technique which are some of the popular techniques used for region based segmentation and so to extract the important information from the medical imaging modalities. An overview and findings of some of the recent and prominent researches are presented here. Damodharan and Raghavan [10] have presented a neural network based technique for brain tumor detection and classification. In this method, the quality rate is produced separately for segmentation of WM, GM, CSF, and tumor region and claims an accuracy of 83% using neural network based classifier. Alfonse and Salem [11] have presented a technique for automatic classification of brain tumor from MR images using an SVM-based classifier. To improve the accuracy of the classifier, features are extracted using fast Fourier transform (FFT) and reduction of features is performed using Minimal-Redundancy-Maximal-Relevance (MRMR) technique. This technique has obtained an accuracy of 98.9%.

The extraction of the brain tumor requires the separation of the brain MR images to two regions [12]. One region contains the tumor cells of the brain and the second contains the normal brain cells [13]. Zanaty [14] proposed a methodology for brain tumor segmentation based on a hybrid type of approach, combining FCM, seed region growing, and Jaccard similarity coefficient algorithm to measure segmented gray matter and white matter tissues from MR images. This method obtained an average segmentation score S of 90% at the noise level of 3% and 9%, respectively. Kong et al. [7] investigated automatic segmentation of brain tissues from MR images using discriminative clustering and future selection approach. Demirhan et al. [5] presented a new tissue segmentation algorithm using wavelets and neural networks, which claims effective segmentation of brain MR images into the tumor, WM, GM, edema, and CSF. Torheim et al. [15], Guo et al. [1], and Yao et al. [16] presented a technique which employed texture features, wavelet transform, and SVM's algorithm for effective classification of dynamic contrast-enhanced MR images, to handle the nonlinearity of real data and to address different image protocols effectively. Torheim et al. [15] also claim that their proposed technique gives better predictions and improved clinical factors, tumor volume, and tumor stage in comparison with first-order statistical features.

Kumar and Vijayakumar [17] introduced brain tumor segmentation and classification based on principal component analysis (PCA) and radial basis function (RBF) kernel based SVM and claims similarity index of 96.20%, overlap fraction of 95%, and an extra fraction of 0.025%. The classification accuracy to identify tumor type of this method is 94% with total errors detected of 7.5%. Sharma et al. [18] have presented a highly efficient technique which claims accuracy of 100% in the classification of brain tumor from MR images. This method is utilizing texture-primitive features with artificial neural network (ANN) as segmentation and classifier tool. Cui et al. [19] applied a localized fuzzy clustering with spatial information to form an objective of medical image segmentation and bias field estimation for brain MR images. In this method, authors use Jaccard similarity index as a measurement of the segmentation accuracy and claim 83% to 95% accuracy to segment white matter, gray matter, and cerebrospinal fluid. Wang et al. [20] have presented a medical image segmentation technique based on active contour model to deal with the problem of intensity inhomogeneities in image segmentation. Chaddad [21] has proposed a technique of automatic feature extraction for brain tumor detection based on Gaussian mixture model (GMM) using MR images. In this method, using principal component analysis (PCA) and wavelet based features, the performance of the GMM feature extraction is enhanced. An accuracy of 97.05% for the T1-weighted and T2-weighted and 94.11% for FLAIR-weighted MR images are obtained.

Deepa and Arunadevi [22] have proposed a technique of extreme learning machine for classification of brain tumor from 3D MR images. This method obtained an accuracy of 93.2%, the sensitivity of 91.6%, and specificity of 97.8%. Sachdeva et al. [23] have presented a multiclass brain tumor classification, segmentation, and feature extraction performed using a dataset of 428 MR images. In this method, authors used ANN and then PCA-ANN and observed the increment in classification accuracy from 77% to 91%.

The above literature survey has revealed that some of the techniques are invented to obtain segmentation only; some of the techniques are invented to obtain feature extraction and some of the techniques are invented to obtain classification only. Feature extraction and reduction of feature vectors for effective segmentation of WM, GM, CSF, and infected tumor region and analysis on combined approach could not be conducted in all the published literature. Moreover, only a few features are extracted and therefore very low accuracy in tumor detection has been obtained. Also, all the above literatures are missing with the calculation of overlap that is dice similarity index, which is one of the important parameters to judge the accuracy of any brain tumor segmentation algorithm.

In this study, we perform a combination of biologically inspired Berkeley wavelet transformation (BWT) and SVM as a classifier tool to improve diagnostic accuracy. The cause of this study is to extract information from the segmented tumor region and classify healthy and infected tumor tissues for a large database of medical images. Our results lead to conclude that the proposed method is suitable to integrate clinical decision support systems for primary screening and diagnosis by the radiologists or clinical experts.

## 3. Materials and Methods

**Figure 1:**
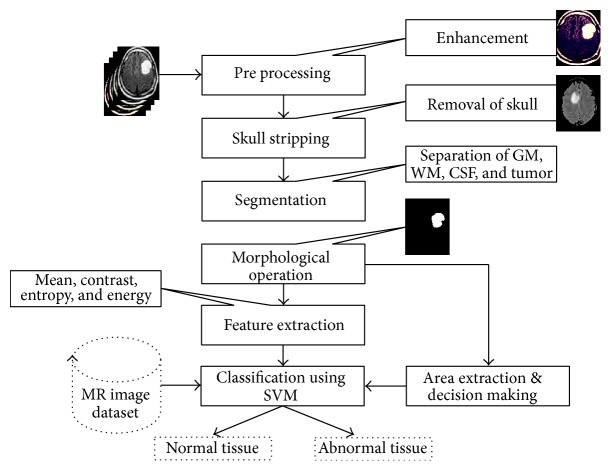
Steps used in proposed algorithm.

This section presents the materials, the source of brain MR image dataset, and the algorithm used to perform brain MR tissue segmentation.  Figure 1 provides the flow diagram of the algorithm. As test images, different MR images of the brain were used, including T1-weighted MR images with Repetition Time (TR) of 1740 and Echo Time (TE) of 20, T2-weighted MR images with Repetition Time (TR) of 5850 and Echo Time (TE) of 130, and FLAIR-weighted MR images with Repetition Time (TR) of 8500 and Echo Time (TE) of 130. These test images were acquired using a 3 Tesla Siemens Magnetom Spectra MR machine. The total numbers of slices for all channels were 15, which leads to total of 135 images at 9 slices or images per patient with a field of view of 200 mm, an interslice gap of 1 mm, and voxel of size 0.78 mm × 0.78 mm × 0.5 mm. The proposed methodology is applied to real dataset including brain MR images of 512 × 512 pixel size and was converted into grayscale before further processing. The following sections discuss the implementation of the algorithm.

### 3.1. Preprocessing

The primary task of preprocessing is to improve the quality of the MR images and make it in a form suited for further processing by human or machine vision system. In addition, preprocessing helps to improve certain parameters of MR images such as improving the signal-to-noise ratio, enhancing the visual appearance of MR image, removing the irrelevant noise and undesired parts in the background, smoothing the inner part of the region, and preserving its edges [5]. To improve the signal-to-noise ratio, and thus the clarity of the raw MR images, we applied adaptive contrast enhancement based on modified sigmoid function [24].

### 3.2. Skull Stripping

**Figure 2:**
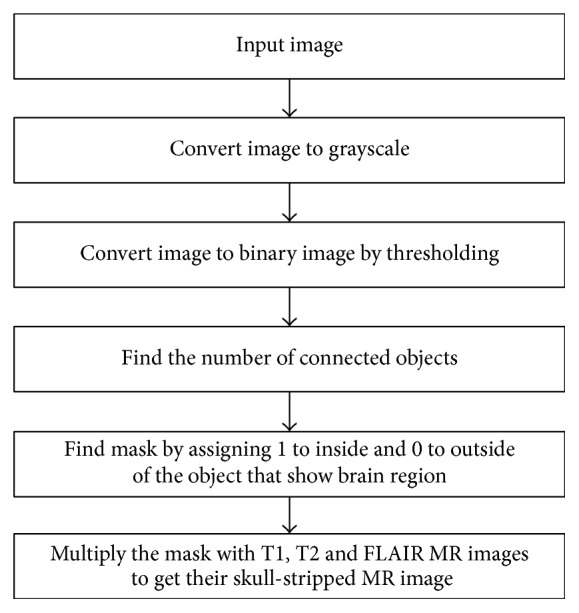
Steps used in the skull stripping algorithm.

Skull stripping is an important process in biomedical image analysis, and it is required for the effective examination of brain tumor from the MR images [25–28]. Skull stripping is the process of eliminating all nonbrain tissues in the brain images. By skull stripping, it is possible to remove additional cerebral tissues such as fat, skin, and skull in the brain images. There are several techniques available for skull stripping; some of the popular techniques are automatic skull stripping using image contour, skull stripping based on segmentation and morphological operation, and skull stripping based on histogram analysis or a threshold value.  Figure 2 provides the stages of the skull stripping algorithm. This study uses the skull stripping technique that is based on a threshold operation to remove skull tissues.

### 3.3. Segmentation and Morphological Operation

The segmentation of the infected brain MR regions is achieved through the following steps: In the first step, the preprocessed brain MR image is converted into a binary image with a threshold for the cut-off of 128 being selected. The pixel values greater than the selected threshold are mapped to white, while others are marked as black; due to this two, different regions are formed around the infected tumor tissues, which is cropped out. In the second step, in order to eliminate white pixel, an erosion operation of morphology is employed. Finally, the eroded region and the original image are both divided into two equal regions and the black pixel region extracted from the erode operation is counted as a brain MR image mask. In this study, Berkeley wavelet transformation is employed for effective segmentation of brain MR image.

A wavelet is a function that is defined over a finite interval of time and has an average value of zero. The wavelet transformation technique is employed to develop functions, operators, data, or information into components of different frequency, which enables studying each component separately. All wavelets are generated from a basic wavelet Ψ(*t*) by using the scaling and translation process defined by (1); a basic wavelet is also referred to as a mother wavelet because it is the point of origin for other wavelets.

(1) $$\begin{array}{c} \Psi_{s,\tau }=\frac{1}{\sqrt{s}}\Psi \left(\;\frac{t-\tau }{s}\;\right), \end{array}$$

where *s* and *τ* are the scale and translation factors, respectively.

The Berkeley wavelet transform (BWT) [29, 30] is described as a two-dimensional triadic wavelet transform and can be used to process the signal or image. Just like the mother wavelet transformation or other families of wavelet transformation, the BWT algorithm will also perform data conversion from a spatial form into temporal domain frequency. The BWT presents an effective way of representation of image transformation and it is a complete orthonormal [30]. The mother wavelet transformation *β*_*θ*_^*φ*^ is piecewise constant function [29, 31]. The substitute wavelets from the mother wavelet *β*_*θ*_^*φ*^ are produced at various pixels positions in the two-dimensional plane through scaling and translation of the mother wavelet and it is shown in

(2) $$\begin{array}{c} \beta_{\theta }^{\varphi }\left(\;\tau ,s\;\right)=\frac{1}{s^{2}}\beta_{x}^{\varphi }\left(\;3^{s}\left(\;x-i\;\right),3^{s}\left(\;y-j\;\right)\;\right), \end{array}$$

where *τ* and *s* are translation and scale parameter of the wavelet transformation, respectively, and *β*_*θ*_^*φ*^ is the transforming function, and it is called the mother wavelet of Berkeley wavelet transformation. The only single constant term is sufficient to represent the mean value of an image; the coefficient value of the single term is shown in

(3) $$\begin{array}{c} \beta_{0}=\frac{1}{\sqrt{9}}\left[\;u\left(\;\frac{x}{3},\frac{y}{3}\;\right)\;\right]. \end{array}$$

The morphological operation is used for the extraction of the boundary areas of the brain images. Conceptually, the morphological operation is only rearranging the relative order of pixel values, not on their mathematical values, and so is suitable to process only binary images. Dilation and erosion are the two most basic operations of morphology. Dilation operations are intended to add pixels to the boundary region of the object, while erosion operations are intended to remove the pixels from the boundary region of the objects. The operation of addition and removing pixels to or from boundary region of the objects is based on the structuring element of the selected image.

**Figure 3:**
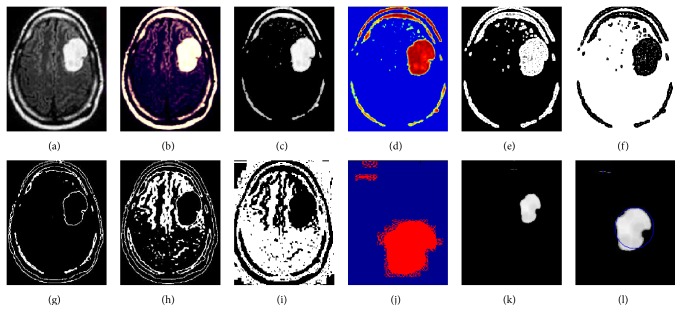
Segmented and area extracted result of brain MR image. (a) Original image. (b) Enhanced image. (c) Skull-stripped image. (d) Wavelet transpose image. (e) Intense segmented image. (f) Inverse intense image. (g) Gray matter. (h) White matter. (i) CSF. (j) Dice overlap image. (k) Eroded image. (l) Area extracted image.

The experimented results produced by the proposed technique depicted for the segmented outcome for the three classes of WM, GM, and CSF and for the extracted tumor region are given in Figure 3. The experimental results also find dice overlap image, indicating the comparison between the algorithm output and ground truth.

### 3.4. Feature Extraction

It is the process of collecting higher-level information of an image such as shape, texture, color, and contrast. In fact, texture analysis is an important parameter of human visual perception and machine learning system. It is used effectively to improve the accuracy of diagnosis system by selecting prominent features. Haralick et al. [32] introduced one of the most widely used image analysis applications of Gray Level Cooccurrence Matrix (GLCM) and texture feature. This technique follows two steps for feature extraction from the medical images. In the first step, the GLCM is computed, and in the other step, the texture features based on the GLCM are calculated. Due to the intricate structure of diversified tissues such as WM, GM, and CSF in the brain MR images, extraction of relevant features is an essential task. Textural findings and analysis could improve the diagnosis, different stages of the tumor (tumor staging), and therapy response assessment. The statistics feature formula for some of the useful features is listed below.

*(1) Mean (M)*. The mean of an image is calculated by adding all the pixel values of an image divided by the total number of pixels in an image.

(4) $$\begin{array}{c} M=\left(\;\frac{1}{m\times n}\;\right)\overset{m-1}{\underset{x=0}{\sum }}\;\overset{n-1}{\underset{y=0}{\sum }}f\left(\;x,y\;\right). \end{array}$$

*(2) Standard Deviation (SD)*. The standard deviation is the second central moment describing probability distribution of an observed population and can serve as a measure of inhomogeneity. A higher value indicates better intensity level and high contrast of edges of an image.

(5) $$\begin{array}{c} SD\left(\;\sigma \;\right)=\sqrt{\left(\frac{1}{m\times n}\right)\overset{m-1}{\underset{x=0}{\sum }}\;\overset{n-1}{\underset{y=0}{\sum }}{\left(f\left(x,y\right)-M\right)}^{2}}. \end{array}$$

*(3) Entropy (E)*. Entropy is calculated to characterize the randomness of the textural image and is defined as

(6) $$\begin{array}{c} E=-\overset{m-1}{\underset{x=0}{\sum }}\;\overset{n-1}{\underset{y=0}{\sum }}f\left(\;x,y\;\right)\log_{2}f\left(\;x,y\;\right). \end{array}$$

*(4) Skewness (S*_*k*_). Skewness is a measure of symmetry or the lack of symmetry. The skewness of a random variable *X* is denoted as *S*_*k*_(*X*) and it is defined as

(7) $$\begin{array}{c} S_{k}\left(\;X\;\right)=\left(\;\frac{1}{m\times n}\;\right)\frac{\sum \left.{\left(f\left(x,y\right)-M\right)}^{3}\right|}{{SD}^{3}}. \end{array}$$

*(5) Kurtosis (S*_*k*_). The shape of a random variable's probability distribution is described by the parameter called Kurtosis. For the random variable *X*, the Kurtosis is denoted as *K*_urt_(*X*) and it is defined as

(8) $$\begin{array}{c} K_{urt}\left(\;X\;\right)=\left(\;\frac{1}{m\times n}\;\right)\frac{\sum \left.{\left(f\left(x,y\right)-M\right)}^{4}\right|}{{SD}^{4}}. \end{array}$$

*(6) Energy (En)*. Energy can be defined as the quantifiable amount of the extent of pixel pair repetitions. Energy is a parameter to measure the similarity of an image. If energy is defined by Haralicks GLCM feature, then it is also referred to as angular second moment, and it is defined as

(9) $$\begin{array}{c} En=\sqrt{\overset{m-1}{\underset{x=0}{\sum }}\;\overset{n-1}{\underset{y=0}{\sum }}f^{2}\left(x,y\right)}. \end{array}$$

*(7) Contrast (C*_*on*_). Contrast is a measure of intensity of a pixel and its neighbor over the image, and it is defined as

(10) $$\begin{array}{c} C_{on}=\overset{m-1}{\underset{x=0}{\sum }}\;\overset{n-1}{\underset{y=0}{\sum }}{\left(\;x-y\;\right)}^{2}f\left(\;x,y\;\right). \end{array}$$

*(8) Inverse Difference Moment (IDM) or Homogeneity*. Inverse Difference Moment is a measure of the local homogeneity of an image. IDM may have a single or a range of values so as to determine whether the image is textured or nontextured.

(11) $$\begin{array}{c} IDM=\overset{m-1}{\underset{x=0}{\sum }}\;\overset{n-1}{\underset{y=0}{\sum }}\frac{1}{1+{\left(x-y\right)}^{2}}f\left(\;x,y\;\right). \end{array}$$

*(9) Directional Moment (DM)*. Directional moment is a textural property of the image calculated by considering the alignment of the image as a measure in terms of the angle and it is defined as

(12) $$\begin{array}{c} DM=\overset{m-1}{\underset{x=0}{\sum }}\;\overset{n-1}{\underset{y=0}{\sum }}f\left(\;x,y\;\right)\left|\;x-y\;\right|. \end{array}$$

*(10) Correlation (C*_*orr*_). Correlation feature describes the spatial dependencies between the pixels and it is defined as

(13) $$\begin{array}{c} C_{orr}=\frac{\sum_{x=0}^{m-1}\sum_{y=0}^{n-1}\left(x,y\right)f\left(x,y\right)-M_{x}M_{y}}{\sigma_{x}\sigma_{y}}, \end{array}$$

where *M*_*x*_ and *σ*_*x*_ are the mean and standard deviation in the horizontal spatial domain and *M*_*y*_ and *σ*_*y*_ are the mean and standard deviation in the vertical spatial domain.

*(11) Coarseness (C*_*ness*_). Coarseness is a measure of roughness in the textural analysis of an image. For a fixed window size a texture with a smaller number of texture elements is said to be more coarse than the one with a larger number. The rougher texture means higher coarseness value. Fine textures have smaller values of coarseness. It is defined as

(14) $$\begin{array}{c} C_{ness}=\frac{1}{2^{m+n}}\overset{m-1}{\underset{x=0}{\sum }}\;\overset{n-1}{\underset{y=0}{\sum }}f\left(\;x,y\;\right). \end{array}$$

Apart from the above textural feature extraction, the following quality assessment parameters are also needed to ensure better result analysis on brain MR images.

*(1) Structured Similarity Index (SSIM)*. The Structural Similarity Index (SSIM) is a perceptual metric that signifies that the degradation in image quality may be caused by data compression or losses in data transmission or by any other means of the image processing. It is defined as

(15) SSIMσxyσxσy2xy¯x−2+y−2+C1·2σxσyσx2+σy2+C2.

A Higher value of SSIM indicates better preservation of luminance, contrast, and structural content.

*(2) Mean Square Error (MSE)*. Mean square error is a measure of signal fidelity or image fidelity. The purpose of signal or image fidelity measure is to find the similarity or fidelity between two images by providing the quantitative score. When MSE is calculated, then it is assumed that one of the images is pristine original, while the other is distorted or processed by some means and it is defined as

(16) $$\begin{array}{c} MSE=\frac{1}{M\times N}\sum \sum {\left(\;f\left(\;x,y\;\right)-f^{R}\left(\;x,y\;\right)\;\right)}^{2}. \end{array}$$

*(3) Peak Signal-to-Noise Ratio (PSNR) in dB*. Peak signal-to-noise ratio is a measure used to assess the quality of reconstruction of processed image and it is defined as

(17) $$\begin{array}{c} \text{PSBR in dB}=20\;\log_{10}\frac{\left(2^{n}-1\right)}{MSE}. \end{array}$$

Lower value of MSE and higher value of PSNR indicate better signal-to-noise ratio.

*(4) Dice Coefficient*. Dice coefficient or dice similarity index is a measure of overlap between the two images and it is defined as

(18) $$\begin{array}{c} Dice\left(\;A,B\;\right)=2\times \frac{\left|A_{1}\wedge B_{1}\right|}{\left(\left|A_{1}\right|+\left|B_{1}\right|\right)}, \end{array}$$

where *A* ∈ {0,1} is tumor region extracted from algorithmic predictions and *B* ∈ {0,1} is the experts ground truth. The minimum value of dice coefficient is 0 and the maximum is 1; a higher value indicates better overlap between the two images.

Tables 1 and 2 show some of the prominent features for the first-order statistical and second-order statistical analysis.  Table 2 also indicates the measure of coarseness and number of key values present in the segmented image.

### 3.5. Support Vector Machine (SVM)

The original SVM algorithm was contributed by Vladimir N. Vapnik and its modern version was developed by Cortes and Vapnik in 1993 [33]. The SVM algorithm is based on the study of a supervised learning technique and is applied to one-class classification problem to n-class classification problems [1, 34–36]. The principle aim of the SVM algorithm is to transform a nonlinear dividing objective into a linear transformation using a function called SVM's kernel function. In this study, we used the Gaussian kernel function for transformation. By using a kernel function, the nonlinear samples can be transformed into a high-dimensional future space where the separation of nonlinear samples or data might become possible, making the classification convenient [16]. The SVM algorithm defines a hyperplane that is divided into two training classes as defined in

(19) $$\begin{array}{c} f\left(\;y\;\right)=Z^{T}\phi \left(\;y\;\right)+b, \end{array}$$

where *Z* and *T* are hyperplane parameters and *ϕ*(*y*) is a function used to map vector *y* into a higher-dimensional space. Equation (20) provides the Gaussian kernel function of nonlinear SVM [16, 34] used for the optimal solution of classification and generalization and its advanced classification function is shown in (21):

(20) kyi,yj=exp−γyi−yj2,

(21) kyi,yj=∑i=1N ∑Xi∈Mjexp−γyi−yj2,

where *y*_*i*_ and *y*_*j*_ are objects *i* and *j*, respectively, and *γ* is a contour parameter used to determine the smoothness of the boundary region [4, 15].

The features selection with kernel class separability makes SVM the default choice for classification of a brain tumor. The SVM algorithm's performance can be evaluated in terms of accuracy, sensitivity, and specificity. The confusion matrix defining the terms TP, TN, FP, and FN from the expected outcome and ground truth result for the calculation of accuracy, sensitivity, and specificity are shown in Table 3.

Where TP is the number of true positives, which is used to indicate the total number of abnormal cases correctly classified, TN is the number of true negatives, which is used to indicate normal cases correctly classified; FP is the number of false positive, and it is used to indicate wrongly detected or classified abnormal cases; when they are actually normal cases and FN is the number of false negatives, it is used to indicate wrongly classified or detected normal cases; when they are actually abnormal cases [15], all of these outcome parameters are calculated using the total number of samples examined for the detection of the tumor. The quality rate parameter accuracy is the proportion of total correctly classified cases that are abnormally classified as abnormal and normally classified as normal from the total number of cases examined [37, 38].  Table 4 shows the formulas to calculate accuracy, sensitivity, and specificity.

## 4. Results and Discussion

To validate the performance of our algorithm, we used two benchmark datasets and one dataset collected from expert radiologists, which included sample images of 15 patients with 9 slices for each patient. The first dataset is the Digital Imaging and Communications in Medicine (DICOM) dataset [39]. For the purpose of the analysis, we considered 22 images from the DICOM dataset, all of which included are tumor-infected brain tissues. However, this dataset did not have any ground truth images. The second dataset is the Brain Web dataset [40], which consists of full three-dimensional simulated brain MR data obtained using three sequences of modalities, namely, T1-weighted MRI, T2-weighted MRI, and proton density-weighted MRI. This dataset included a variety of slice thicknesses, noise levels, and levels of intensity nonuniformity. The images used for our analysis are mostly included T2-weighted modality with 1 mm slice thickness, 3% noise, and 20% intensity nonuniformity. In this dataset, 13 out of 44 images included are tumor-infected brain tissues. The last dataset collected from expert radiologists consisted of 135 images of 15 patients with all modalities. This dataset had ground truth images that helped to compare the results of our method with the manual analysis of radiologists.

**Figure 4:**
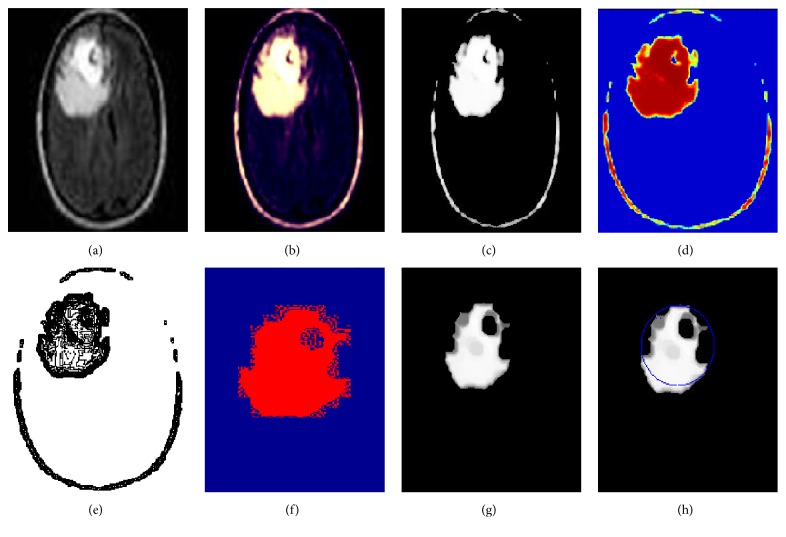
Experimental results of image 1. (a) Original image. (b) Enhanced image. (c) Skull-stripped image. (d) Wavelet decompose image. (e) Intense segmented image. (f) Dice overlap image. (g) Tumor region. (h) Area extracted tumor region.

**Figure 5:**
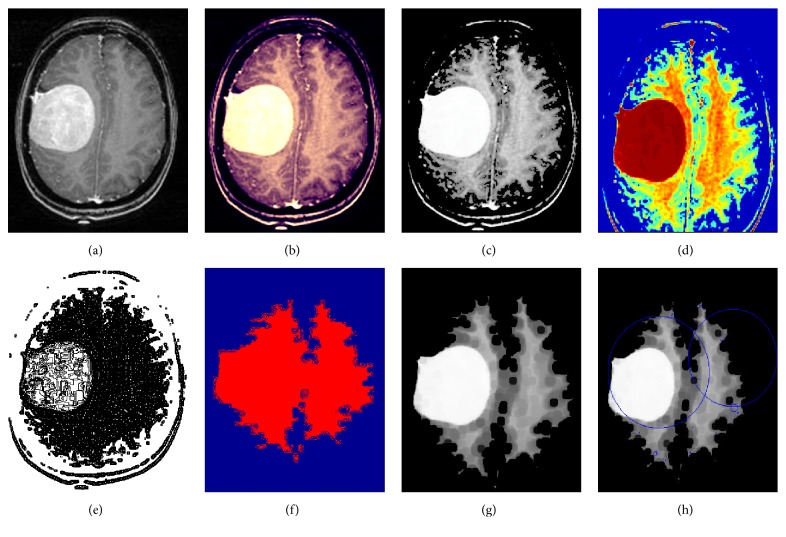
Experimental results of image 2. (a) Original image. (b) Enhanced image. (c) Skull-stripped image. (d) Wavelet decompose image. (e) Intense segmented image. (f) Dice overlap image. (g) Tumor region. (h) Area extracted tumor region.

**Figure 6:**
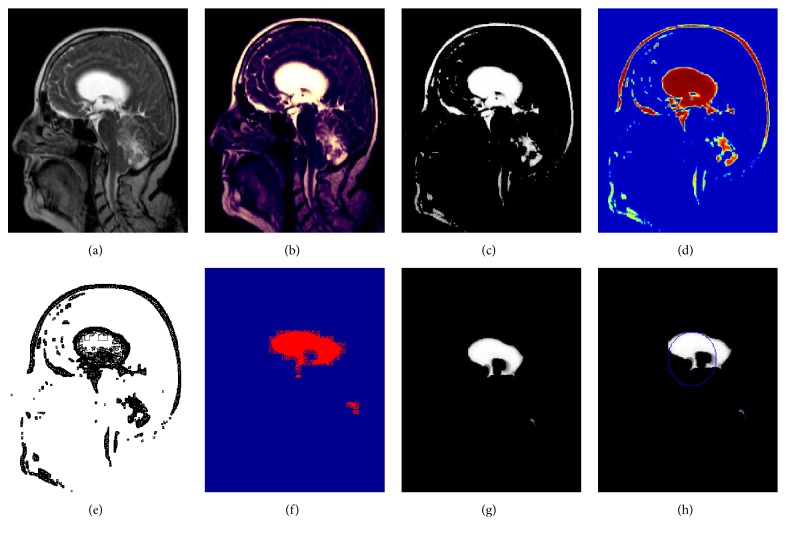
Experimental results of image 3. (a) Original image. (b) Enhanced image. (c) Skull-stripped image. (d) Wavelet decompose image. (e) Intense segmented image. (f) Dice overlap image. (g) Tumor region. (h) Area extracted tumor region.

This section presents the results of our proposed image segmentation technique, which are obtained by using real brain MR images. The proposed algorithm was carried out using Matlab 7.12.0 (R2011a), which runs on the Windows 8 operating system and has an Intel core i3 processor and a 4 GB RAM. The sample experimental results obtained from the proposed technique that are depicted in Figures 4, 5, and 6 show the original image along with enhanced image, skull-stripped image, wavelet decompose image, cluster (intense) segmented image, dice overlap image, and the tumor region with extracted area mark.

Table 5 provides the details of the different performance parameters such as mean squared error (MSE) and peak signal-to-noise ratio (PSNR), structured similarity index (SSIM), and dice score. A lower value of MSE and a higher value of PSNR indicate better signal-to-noise ratio in the extracted image. Dice coefficient measures the overlap of the automatic and manual segmentation for the given dataset. It is important to note that as some of the features do not contribute to the classification, it is around 86.14% in an adaptive fuzzy inference system (ANFIS), 80.29% in Back Propagation, 90.54% in SVM, and 84.55% in *K*-Nearest Neighbors (*K*-NN) without feature extraction.  Table 6 shows the accuracy of the classification without feature extraction and with feature extraction and shows that it will increase the performance of the classifiers on the diagnosis of the tumor from brain MR image with feature extraction. The test performance of the SVM classifier determined by the computation of the statistical parameters such as sensitivity, specificity, and accuracy in comparison with different classifier techniques is shown in Table 7. Furthermore, higher values of accuracy and sensitivity and a lower value of specificity indicate better performance. It can be seen from Table 7 that the performance of our segmentation algorithm is better than the state-of-the-art techniques. Even a modest improvement in the sensitivity parameter is very important and critical for a radiologist or clinical doctors for surgical planning.

The proposed algorithm performs segmentation, feature extraction, and classification as is done in human vision perception, which recognizes different objects, different textures, contrast, brightness, and depth of the image. Moreover, if certain agents are applied effectively, the application of the proposed technique can be extended to a varying range of tumors and MR modalities. In a future study, we intend to investigate the application of the proposed method to more realistic and more clinically bounded cases with a large variety of scenarios covering different aspects by using large dataset.  Table 8 shows the area of the extracted brain tumor in square cm and pixels and its comparison with the area calculated by expert radiologists.

## 5. Comparative Analysis

**Figure 7:**
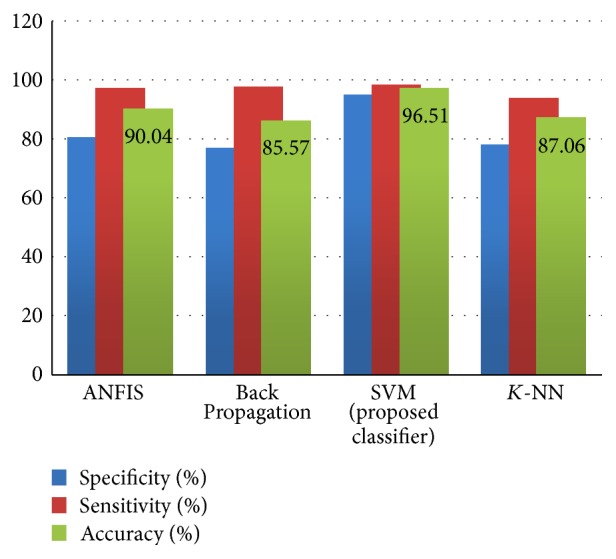
Comparative analysis of classifiers.

The result obtained using the proposed brain tumor detection technique based on Berkeley wavelet transform (BWT) and support vector machine (SVM) classifier is compared with the ANFIS, Back Propagation, and *K*-NN classifier on the basis of performance measure such as sensitivity, specificity, and accuracy. The detailed analysis of performance measures is shown in Figure 7 and, through the performance measure, it is depicted that the performance of the proposed methodology has significantly improved the tumor identification compared with the ANFIS, Back Propagation, and *K*-NN based classification techniques.

## 6. Conclusion and Future Work

In this study, using MR images of the brain, we segmented brain tissues into normal tissues such as white matter, gray matter, cerebrospinal fluid (background), and tumor-infected tissues. Fifteen patients infected with a glial tumor, in benign and malignant stages, assisted in this study. We used preprocessing to improve the signal-to-noise ratio and to eliminate the effect of unwanted noise. We used a skull stripping algorithm based on threshold technique to improve the skull stripping performance. Furthermore, we used Berkeley wavelet transform to segment the images and support vector machine to classify the tumor stage by analyzing feature vectors and area of the tumor. In this study, we investigated texture based and histogram based features with a commonly recognized classifier for the classification of brain tumor from MR brain images. From the experimental results performed on the different images, it is clear that the analysis for the brain tumor detection is fast and accurate when compared with the manual detection performed by radiologists or clinical experts. The various performance factors also indicate that the proposed algorithm provides better result by improving certain parameters such as mean, MSE, PSNR, accuracy, sensitivity, specificity, and dice coefficient. Our experimental results show that the proposed approach can aid in the accurate and timely detection of brain tumor along with the identification of its exact location. Thus, the proposed approach is significant for brain tumor detection from MR images.

The experimental results achieved 96.51% accuracy demonstrating the effectiveness of the proposed technique for identifying normal and abnormal tissues from MR images. Our results lead to the conclusion that the proposed method is suitable for integrating clinical decision support systems for primary screening and diagnosis by the radiologists or clinical experts.

In the future work, to improve the accuracy of the classification of the present work, we are planning to investigate the selective scheme of the classifier by combining more than one classifier and feature selection techniques.

## Figures and Tables

**Table 1:** First-order statistical features for few images.

Images	Mean	Standard deviation	Skewness	Kurtosis	Energy	Entropy
Image 1	8.66	43.99	0.00553	2.89041*E* − 06	10.94	0.65
Image 2	11.81	49.11	0.00655	2.74079*E* − 06	16.37	0.94
Image 3	39.40	75.59	0.01054	1.8506*E* − 06	65.99	3.03
Image 4	6.83	39.45	0.00517	3.33685*E* − 06	8.11	0.45
Image 5	11.90	38.81	0.02002	1.35422*E* − 05	33.17	2.09
Image 6	5.33	28.95	0.01647	2.05493*E* − 05	13.87	1.12

**Table 2:** Second-order textural features with coarseness and key points for few images.

Images	Contrast	Homogeneity	Energy	Correlation	Coarseness	Key points
Image 1	0.2659	0.9253	0.4088	0.9856	8.85	2202
Image 2	0.4735	0.8633	0.3823	0.9458	11.77	932
Image 3	0.2766	0.9323	0.6936	0.9456	13.65	1755
Image 4	0.3569	0.8984	0.3481	0.9773	16.91	1736
Image 5	0.3341	0.8985	0.2660	0.9835	13.52	1540
Image 6	0.3042	0.9038	0.3843	0.9808	14.70	1205

**Table 3:** Confusion matrix defining the terms TP, TN, FP, and FN.

Expected outcome	Ground truth		Row total
	Positive	Negative	
Positive	TP	FP	TP + FP
Negative	FN	TN	FN + TN
Column total	TP + FN	FP + TN	TP + FP + FN + TN

**Table 4:** Accuracy, sensitivity, and specificity calculation.

Quality parameter	Formula
Accuracy	$\frac{TP+TN}{TP+TN+FP+FN}$
Sensitivity	$\frac{TP}{TP+FN}$
Specificity	$\frac{TN}{TN+FP}$

**Table 5:** Performance analysis parameters for segmented tissues.

Images	MSE	PSNR	SSIM	Dice score
Image 1	1.86	55.45 dB	0.8944	0.83
Image 2	0.58	68.21 dB	0.9025	0.87
Image 3	4.95	56.28 dB	0.9702	0.82
Image 4	1.23	58.79 dB	0.8801	0.79
Image 5	5.06	59.65 dB	0.7978	0.90

**Table 6:** Classification accuracies based on feature extraction.

Classifiers	Accuracy (%) without feature extraction	Accuracy (%) with feature extraction
ANFIS	86.14	90.04
Back Propagation	80.29	85.57
SVM (proposed classifier)	90.54	96.51
*K*-NN	84.55	87.06

**Table 7:** Comparison of accuracies in different classifiers.

Number of test images (normal = 67, abnormal = 134)				
Evaluation parameter	ANFIS	Back Propagation	Proposed classifier (SVM)	*K*-NN
True negative	63	62	65	63
False positive	16	19	4	18
True positive	118	110	129	112
False negative	4	10	3	8
Specificity (%)	79.74	76.54	94.2	77.77
Sensitivity (%)	96.72	97.5	97.72	93.33
Accuracy (%)	90.04	85.57	96.51	87.06

**Table 8:** Area of the extracted tumor.

Images	Original image size	Area in pixel	Area of extracted tumor	Area in square centimeters	Area ratio	Accuracy of the area compared to the area calculated by expert radiologist
Image 1	274 × 278	76172	9877	1.22	0.1296	99.8%
Image 2	257 × 256	65792	7064	0.58	0.1073	100%
Image 3	336 × 407	136752	6365	1.45	0.0465	100%
Image 4	200 × 198	39600	7608	0.23	0.1921	99.8 %
Image 5	336 × 204	68544	4494	1.79	0.1079	100%

## References

[1] Guo L., Zhao L., Wu Y., Li Y., Xu G., Yan Q. (2011). Tumor detection in MR images using one-class immune feature weighted SVMs. *IEEE Transactions on Magnetics*.

[2] Kumari R. (2013). SVM classification an approach on detecting abnormality in brain MRI images. *International Journal of Engineering Research and Applications*.

[3] American Brain Tumor Association http://www.abta.org.

[4] Gordillo N., Montseny E., Sobrevilla P. (2013). State of the art survey on MRI brain tumor segmentation. *Magnetic Resonance Imaging*.

[5] Demirhan A., Toru M., Guler I. (2015). Segmentation of tumor and edema along with healthy tissues of brain using wavelets and neural networks. *IEEE Journal of Biomedical and Health Informatics*.

[6] Madhukumar S., Santhiyakumari N. (2015). Evaluation of k-Means and fuzzy C-means segmentation on MR images of brain. *Egyptian Journal of Radiology and Nuclear Medicine*.

[7] Kong Y., Deng Y., Dai Q. (2015). Discriminative clustering and feature selection for brain MRI segmentation. *IEEE Signal Processing Letters*.

[8] El-Melegy M. T., Mokhtar H. M. (2014). Tumor segmentation in brain MRI using a fuzzy approach with class center priors. *EURASIP Journal on Image and Video Processing*.

[9] Coatrieux G., Huang H., Shu H., Luo L., Roux C. (2013). A watermarking-based medical image integrity control system and an image moment signature for tampering characterization. *IEEE Journal of Biomedical and Health Informatics*.

[10] Damodharan S., Raghavan D. (2015). Combining tissue segmentation and neural network for brain tumor detection. *International Arab Journal of Information Technology*.

[11] Alfonse M., Salem A.-B. M. (2016). An automatic classification of brain tumors through MRI using support vector machine. *Egyptian Computer Science Journal*.

[12] Ain Q., Jaffar M. A., Choi T.-S. (2014). Fuzzy anisotropic diffusion based segmentation and texture based ensemble classification of brain tumor. *Applied Soft Computing Journal*.

[13] Abdel-Maksoud E., Elmogy M., Al-Awadi R. (2014). Brain tumor segmentation based on a hybrid clustering technique. *Egyptian Informatics Journal*.

[14] Zanaty E. A. (2012). Determination of gray matter (GM) and white matter (WM) volume in brain magnetic resonance images (MRI). *International Journal of Computer Applications*.

[15] Torheim T., Malinen E., Kvaal K. (2014). Classification of dynamic contrast enhanced MR images of cervical cancers using texture analysis and support vector machines. *IEEE Transactions on Medical Imaging*.

[16] Yao J., Chen J., Chow C. (2009). Breast tumor analysis in dynamic contrast enhanced MRI using texture features and wavelet transform. *IEEE Journal on Selected Topics in Signal Processing*.

[17] Kumar P., Vijayakumar B. (2015). Brain tumour Mr image segmentation and classification using by PCA and RBF kernel based support vector machine. *Middle-East Journal of Scientific Research*.

[18] Sharma N., Ray A., Sharma S., Shukla K., Pradhan S., Aggarwal L. (2008). Segmentation and classification of medical images using texture-primitive features: application of BAM-type artificial neural network. *Journal of Medical Physics*.

[19] Cui W., Wang Y., Fan Y., Feng Y., Lei T. (2013). Localized FCM clustering with spatial information for medical image segmentation and bias field estimation. *International Journal of Biomedical Imaging*.

[20] Wang G., Xu J., Dong Q., Pan Z. (2014). Active contour model coupling with higher order diffusion for medical image segmentation. *International Journal of Biomedical Imaging*.

[21] Chaddad A. (2015). Automated feature extraction in brain tumor by magnetic resonance imaging using gaussian mixture models. *International Journal of Biomedical Imaging*.

[22] Deepa S. N., Arunadevi B. (2013). Extreme learning machine for classification of brain tumor in 3D MR images. *Informatologia*.

[23] Sachdeva J., Kumar V., Gupta I., Khandelwal N., Ahuja C. K. (2013). Segmentation, feature extraction, and multiclass brain tumor classification. *Journal of Digital Imaging*.

[24] Lal S., Chandra M. (2014). Efficient algorithm for contrast enhancement of natural images. *International Arab Journal of Information Technology*.

[25] Benson C. C., Lajish V. L. Morphology based enhancement and skull stripping of MRI brain images.

[26] Oo S. Z., Khaing A. S. (2014). Brain tumor detection and segmentation using watershed segmentation and morphological operation. *International Journal of Research in Engineering and Technology*.

[27] Roslan R., Jamil N., Mahmud R. (2011). Skull stripping magnetic resonance images brain images: region growing versus mathematical morphology. *International Journal of Computer Information Systems and Industrial Management Applications*.

[28] Mohsin S., Sajjad S., Malik Z., Abdullah A. H. (2012). Efficient way of skull stripping in MRI to detect brain tumor by applying morphological operations, after detection of false background. *International Journal of Information and Education Technology*.

[29] Willmore B., Prenger R. J., Wu M. C., Gallant J. L. (2008). The Berkeley wavelet transform: a biologically inspired orthogonal wavelet transform. *Neural Computation*.

[30] Remya Ravindran P., Soman K. P. Berkeley wavelet transform based image watermarking.

[31] Alwan I. M., Jamel E. M. (2015). Digital image watermarking using Arnold scrambling and Berkeley wavelet transform. *Al-Khwarizmi Engineering Journal*.

[32] Haralick R. M., Shanmugam K., Dinstein I. (1973). Textural features for image classification. *IEEE Transactions on Systems, Man and Cybernetics*.

[33] Liu J., Li M., Wang J., Wu F., Liu T., Pan Y. (2014). A survey of MRI-based brain tumor segmentation methods. *Tsinghua Science and Technology*.

[34] Nanthagopal A. P., Sukanesh R. (2013). Wavelet statistical texture features-based segmentation and classification of brain computed tomography images. *IET Image Processing*.

[35] Anitha V., Murugavalli S. (2014). Brain tumor classification based on clustered discrete cosine transform in compressed domain. *Journal of Computer Science*.

[36] Parveen, Singh A. Detection of brain tumor in MRI images, using combination of fuzzy c-means and SVM.

[37] Dhanalakshmi K., Rajamani V. (2013). An intelligent mining system for diagnosing medical images using combined texture-histogram features. *International Journal of Imaging Systems and Technology*.

[38] Rajendran P., Madheswaran M. Pruned associative classification technique for the medical image diagnosis system.

[39] DICOM Samples Image Sets, http://www.osirix-viewer.com/

[40] Brain web: Simulated Brain Database. http://brainweb.bic.mni.mcgill.ca/cgi/brainweb1.

